# A Comprehensive Model for Trauma Research Design

**DOI:** 10.5812/atr.5288

**Published:** 2012-06-01

**Authors:** Hamid Honarpisheh

**Affiliations:** 1 Iranian Medical Science Council's Secretariat, Deputy of Education, Ministry of Health and Medical Education, Tehran, IR Iran

**Keywords:** Research Design, Theoretical Model, Trauma Severity Indices, Wounds and Injuries

## Abstract

Concomitant research and education are invaluable for patient care and medical practice in trauma. Elucidation of a foundation for the integration of training and service that can be combined with research in trauma is crucial, and every trauma case should be studied for this purpose. In this study, we investigated the unique features of trauma research to formulate a generic comprehensive model that can be used at any point at which one may desire to develop a research plan. The framework of this model is designed to enable proper trauma research plain in combination with the best routine trauma care. Selection of the appropriate method of study, the corresponding basic questions raised, aims, and the relevant epidemiologic context are factors that are included in this review. Furthermore, suitable sources, proper time for data collection, reliable and valid measures, and criteria for the scaling and quantification of the findings are indicated. In addition, the levels, orders, operational stages, and steps to be taken in planning research projects are logically set based on the principles of cognitive task analysis, and correspond to the entire spectrum of trauma care situations. Lastly, a measure of utility value is assigned in terms of the expected extent of efficiency and presumed level of effectiveness.

## 1. Introduction

Injury has become a major cause of death and disability worldwide (1). Trauma refers to any event or accident and the collection of consequences that occur thereafter, which usually result in physical and mental emergency problems. Impairments, disabilities, and the delayed mental, cognitive, occupational, social, and economic effects of trauma are usually overlooked due to the emergency and critical conditions involved or the urgent care that is needed in most cases. Since traumatic events disrupt the lives of all of the individuals involved, it is necessary that all trauma patients are fully taken care of and well-managed (2). Most of the initial care and medical services delivered after trauma are expected to be emergent and urgent, and no delay is acceptable. However, as in all other fields of medicine, research is a necessary and inevitable endeavor to achieve the desired improvement in the practice of trauma care. All observations, descriptions, interventions, and control of the events in any research setting should be considered in the context of the real world to provide the most desirable environment for research and learning in trauma care practice. 

### 1.1. A Comprehensive Model of Trauma Research

Diagnosis, care, and research in trauma are accounted for in this model. Management of trauma is a matter of art rather than science, as most trauma cases do not follow similar patterns. Natural biological processes of the body and mind eventually are broken in trauma, and for this reason not any single case of trauma research would provide the necessary conditions for a test of predefined hypotheses. Therefore, the proposed model aims to emphasize an evidence-based design comprising 6 sequential steps in 3 parts. It includes the definition of the origin of trauma on the body and the type of trauma, the assessment of the severity of the trauma and extent of the injuries, and finally, the study of the effects of the intervention and formulation of a prospective evidence-based guideline for the prevention and care, as appropriate. The requirement for all these different aspects is probably the reason why trauma research is mainly carried out in trauma centers, trauma research centers, or trauma audit and research networks.

The general trend in the design of a research plan follows the methodologies used in epidemiologic studies. In this model, studies of trauma focus on the case as well as the event; the causal relationship under study is more immediate and the study design and research mentality is meant to be more evidence-based. This model begins with the case and its management as the main event, with the overall goal of progressing to the development of the full evidence-based hypothesis and a long-term research plan (Table 1). **In Table 1 **each level of methodology of research in trauma is characterized by the most appropriate method to be implemented, relevant question(s), design and the epidemiologic context. 

**Table 1.** Levels of Methodology of Trauma Research

Health And Medical Service Management	Epidemiology (Context)	Basic Question	Method	Design
Definition and identification of the case and the event	Sentinel event	What happened?	Descriptive (develop hypothesis)	Case study
Verification of the case and the event	Report new diseases or injuries	Did it happen again and again?	Descriptive (develop hypothesis)	Case series
Clarification, quantification and distribution of the event and propose the evidence-based hypothesis	Measure existing disease and current exposure levels and provide some indication of the relationship between injury and exposure or non-exposure and develop hypothesis	How often does it happen?, to whom?, where?, when?	Descriptive (develop hypothesis)	Epidemiol-ogic study
Diagnose the occurrence of the trauma and the current states of exposure	Identify existing injuries and look back in previous years to identify previous exposures to causal factors	What is the coexisting condition of trauma and exposure in real life at a time section?	Analytic studies (identifying hypotheses)	Ecologic study and cross-sectional study
Identify existing injuries and look back to identify the immediate cause of the event and the injury in the scene	Identify existing injuries and look back in previous years to identify previous exposures to causal factors and analyses examine if exposure levels are different between the groups	Is there any kind of relationship in between, retrospectively? What is the odds ratio and the corresponding chances?	Analytic studies (identifying hypotheses)	Case-crossover study and case-control
Propose hypotheses and formulations for better care and case management	Identify existing exposure levels and track disease as it occurs over time. Identify the existing injuries, define the most appropriate interventions and the existing conditions and follow up any emergent change prospectively.	Would there be any kind of relationship? What is the relative and the attributable risks or chances?	Analytic studies (identifying hypotheses)	Cohort study
Practice in urgent care and emergency medical service setting.	Investigate the situation before and after the best possible intervention or event.	Is there any associated change before and after a given intervention or event?	Interventional study	Before-and-after study
Practice in urgent care and emergency medical service setting looking for the prognoses and the expectations in diagnostic and therapeutic management.	Test the hypotheses and formulations for better care and case management.	Is the associated change identified over a controlled intervention verified?	Interventional study	Randomized controlled trial –RCT

## 2. Type of Trauma and the Site of Injury

### 2.1. Diagnosis of the Site of Injury

Diagnosis of the site of injury as the initial stage of trauma care is of prime importance, and is part of the primary survey or surveillance program that is carried out during or immediately after the resuscitative phase. Data related to the vital signs and the functional capacities of the body should be recorded or kept in mind at this stage. As a secondary survey, advanced resuscitative measures are taken to save lives and prevent of further trauma from occurring. At this stage the involved vital organs, damaged body parts, and impaired capacities for bodily functions should be noticed. A rapid anatomic examination of the body parts at the traumatized site and related functions prone to damage in trauma is of prime significance.

The main data to be recorded in the initial stage after trauma are:

a) The vital organs involved

b) Imminent vital signs

c) Vital signs after resuscitation

d) Damaged body parts 

e) Impaired bodily functional capacities 

f) Status of the body parts prone to damage

Major sites of trauma proposed and some relative incidences reported are listed in *Table 2. *

**Table 2.** Major Sites of Injury and the Reported Relevant Incidences Resulting in Hospitalization

	Relative Incidence, %
Head injury	24–30
Neck trauma	5.7
Spinal cord injury	9.9
Chest trauma	9.5–20
Abdominal trauma	10
Extremity trauma	2–37
Pelvic trauma	9.9
Polytrauma	40

### 2.2. Defining the Type and Mechanisms of Trauma

Types of trauma are defined according to the age, gender, and occupation of the trauma patient. The physiologic reactions and psychological status of the patients after trauma differ significantly in various groups, particularly in pediatric, geriatric, and pregnant patients.

The physical characteristics of the direct object that caused the trauma define the main mechanisms of trauma as blunt, penetrating, or explosive. Most studies of trauma follow this category system due to the different consequences of these types of trauma. Other classification schemes for the mechanisms of trauma are based on the type of immediate events causing the damage in trauma. Although the information related to these events is not taken very seriously, it is important and can be collected by general interviews or by special checklists. These events are classified according to the causal incident and are as follows:

• Motor vehicle traffic accidents

• Fall

• Struck by, against

• Transport, other

• Firearm

• Cut/pierce

• Other specified and classifiable

• Pedal cyclist, other

• Fire/burn

• Machinery

## 3. Assessment of the Severity of Trauma and the Magnitude and Prognosis of Damage

### 3.1. Assessment of the Severity of Trauma and the Magnitude of Damage

Assessment of injury severity is an integral component in injury research and injury control (3, 4). The use of different systems for the assessment of injury severity in quantitative trauma research studies has been quite promising. However, the complexity of many of these systems has restricted their practical application. The main purpose of the use of these systems is the assessment of the severity of trauma and the extent of the injury in terms of quantitative numerical or ordinal parameters. Unlike many other studies regarding trauma, the terms “injury” and “trauma” are not used interchangeably throughout this review article. Thus, a correlation between the severity of trauma and the extent of damage can be studied.

Although the implementation of these inventories renders the clinical states of the patient and the degree of conclusive evaluations less prominent, it improves the level of research methodology and makes research calculations more practical. To increase the level of accuracy of these measures, the anatomical damage, physiological impairments, and the functional reserves of the patient in response to trauma should be determined. The most important point to consider in the design of these methods and inventories is the selection of proper key indicators.

**Figure 1.**
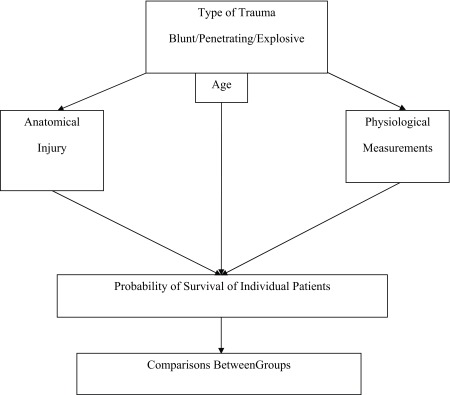
Aspects of Injury Severity

While anatomy, physiology, and host factors may influence the manner in which injury severity is assessed, these variables do not occur in a vacuum. The relevant variables ultimately work together to determine the outcome of a patient care following injury. Importantly, several of the injury severity scales are based only on one aspect of this model (*Figure 1*). 

The most widely used clinical system for the assessment of the state of consciousness as a measure of injury severity is the Glasgow Coma Scale (GCS), which is used in first observation in the physical examination immediately after trauma and during the initial recovery phase (5). It is employed widely as a triage and as a set of prognostic indicators. The parameters of the GCS are the best eye response, the best verbal response, and the best motor response to stimuli, and not just any response. The intensities of the stimuli range from no stimulus (spontaneous responses) to painful stimuli. The character of the response ranges from no response to an oriented verbal response, and that of the motor response varies from nil or reflex responses to obeying commands properly and appropriately. These parameters signify the key indicators. Expression of the GCS assessment as a single number less than 8, from 9 to 12 and above 12 is a rough estimate of severe, moderate, and mild injuries, respectively, and is not sufficiently accurate for the purposes of research in trauma. A more detailed formula indicating the score in the 3 components would be more useful in this respect, for example GCS 11= E3 V4 M4. Of note, pediatric GCS scores differ in terms of the responses *(Table 3)*.

**Table 3.** The Glasgow Coma Scale

	Score	Parameter
Eye opening		
	1	Nil
	2	To pain
	3	To speech
	4	Spontaneously
Motor response		
	1	Nil
	2	Extensor
	3	Flexor
	4	Withdrawal
	5	Localizing
	6	Obeys command
Verbal response		
	1	Nil
	2	Groans
	3	Words
	4	Confused
	5	Oriented

The Organ Injury Scales were developed by the Organ Injury Scaling Committee of the American Association for the Surgery of Trauma (AAST) (6). It provides a common nomenclature by which physicians may describe the sustained injuries and their severity. In this system, the injuries to each organ are assessed specifically and separately by organ, by mechanism (“blunt” vs. “penetrating”) or by anatomic description (“hematoma”, “laceration”, “contusion”, “vascular”). Each organ injury may be graded from 1 to 6; a “1” is assigned to the least severe injury while a “5” is assigned to the most severe injury from which the patient may survive. Grade 6 injuries are, by definition, severe enough to threaten the patient’s life. The clinical condition as the indicator used makes this system very useful in trauma research in the clinical setting. The Abbreviated Injury Scale (AIS) is an anatomical scoring system based on the International Classification of Diseases (ICD-9) that has many similarities to the Organ Injury Scales of the AAST. The AIS is not an injury scale, as the difference between AIS1 and AIS2 is not the same as that between AIS4 and AIS5 (6). In this system, the score assigned is a subjective assessment made by an expert based on 4 criteria implicating threat to life, permanent impairment, treatment period, and energy dissipated. The scoring system is provided in Table 4. 

**Table 4.** Abbreviated Injury Scale (AIS) Injury Scores

Injury	AIS ^a^ Score
Minor	1
Moderate	2
Serious	3
Severe	4
Critical	5
Non-survivable	6

^a^ Abbreviation: AIS, Abbreviated Injury Scale

The Injury Severity Score (ISS) is an anatomical scoring system that provides an overall score for patients with multiple injuries (7). Each injury is assigned an AIS score and is allocated to one of 6 body regions (head, face, chest, abdomen, extremities [including pelvis], and external). Only the highest AIS score in each body region is used. The 3 most severely injured body regions have their scores squared and added together to produce the ISS score. Summing of the squares in this scale provides a greater approximation to mortality prediction (7). An example of the ISS calculation is shown in *Table 5. *

**Table 5.** Injury Severity Scores (ISS) and Descriptions

	Injury Description	AIS ^a^ score	Squared Result of Top 3 Abbreviated Injury Scale (AIS) scores^b^
Head and neck	Cerebral contusion	3	9
Face	No injury	0	
Chest	Flail chest	4	16
Abdomen	Minor contusion of liver	2	
	Complex rupture spleen	5	25
Extremity	Fractured femur	3	
External	No injury	0	

^a^Abbreviation: AIS, Abbreviated Injury Scale

^b^Total Injury Score (n = 50)

The ISS score results in values from 0 to 75. If an injury is assigned an AIS of 6 (non-survivable injury), the ISS score is assigned as 75. The ISS score is virtually the only anatomical scoring system in use that correlates linearly with mortality, morbidity, hospital stay, and other measures of trauma burden (7). No single region can be represented more than once in the score (6). The ISS system is also based on a subjective assessment of severity made by experts and does not differentiate between the injuries of different body regions. The New Injury Severity Score (NISS), which is very similar to the ISS, uses the 3 most severe AIS scores regardless of their body region location. Thus, multiple injuries within the same body region can be considered with the NISS. The following example from an individual with 5 injuries in 4 body regions illustrates the difference between the scales in *(Table 6)*.

**Table 6.** Comparison of the Injury Severity Score (ISS ^a^) and the New Injury Severity Score (NISS ^a^)Scoring of Injuries^a^

	AIS^b^Score	
Multiple abrasions	1	External
Deep laceration tongue	2	Face
Subarachnoid hemorrhage	3	Head/Neck
Major kidney laceration	4	Abdomen
Major liver laceration	4	Abdomen

^a^ISS = (4)^2^ + (3)^2^ + (2)^2^ = 29; NISS = (4)^2^ + (4)^2^ + (3)^2^ = 41

^b^ Abbreviation: AIS, Abbreviated Injury Score

A conversion system relates specific ICD codes to AIS codes; therefore, it is possible to derive ISS and NISS scores from ICD-9-CM Codes. A computer program allows this process to be automated with existing medical datasets. The Anatomic Profile (AP) system, not widely used in injury severity scoring, also uses AIS severity scores, and its measure is made up of four components (labeled A through D). The A, B, and C components represent serious injuries, which correspond to AIS scores of 3 or greater. The AP differs from the ISS (and is similar to the NISS) as it includes multiple injuries within a body region in its assessment (*Table 7*).

The Revised Trauma Score (RTS), the most widely used physiologic measure, is not limited to patients with brain trauma or central nervous system involvement. It provides a scored assessment of the physiology of the individual based upon the values of 3 indicators: respiratory rate (RR), blood pressure, and the GCS; it is the sole value that is documented in record systems upon patient arrival at the hospital for triage decisions or to determine which patients go to Level 1 or Level 2 trauma centers. This assessment may also be used for determining prognosis if the RTS on arrival is compared to the best RTS after resuscitation *(Table 8).*

**Table 7.** The Anatomic Profile of Abbreviated Injury Scale (AIS ^a^) Scaling

Components ^b^	AIS ^a^ Region	AIS ^a^ Severity
A	Head/brain and spinal cord	3–6
B	Thorax, front of neck	3–6
C	AIS region	3–6
D	Head/brain and spinal cord	1–2

^a^Abbreviation: AIS, Abbreviated injury scale

^b^The sum of squares of AIS scores is used to summarize a component’s injuries

**Table 8.** The Revised Trauma Scoring System (RTS).

Clinical Parameter	Category	Score	Weight, kg
Respiratory rate breaths per minute			0.2908
	> 29	4	
	10–29	3	
	6–9	2	
	1–5	1	
	0	0	
Systolic blood pressure			0.7326
	>89	4	
	76–89	3	
	50–75	2	
	1–49	1	
	0	0	
Glasgow coma scale			0.9368
	13–15	4	
	9–12	3	
	6–8	2	
	4–5	1	
	3	0	

Intubation restricts the assessment of verbal responses and RR; therefore, the motor response and eye response of the GCS should be used as estimates of these, respectively, or alternatively, pulse rate or systolic blood pressure (SBP) values should be used alone. When used for outcome analysis (non-triage uses), the scores in each clinical category (RR, blood pressure, GCS) of the RTS are weighted. These values provide more accurate assessments of outcome than the non-weighted RTS, and correlate well with the probability of survival. The value weights are based upon outcome data from the Major Trauma Outcome Study (MTOS) (8). The RTS value is obtained from the following formula:

RTS = 0.9368 GCS + 0.7326 SBP + 0.2908 RR

It is possible to model survival probability following trauma by the use of anatomic measures, physiologic measures, and age in combination. The methods that are predominantly implemented are the Trauma and Injury Severity Score (TRISS) and A Severity Characterization of Trauma (ASCOT) measure. In TRISS the probability of survival is assessed based upon the RTS, mechanism of injury (blunt/penetrating), age, and ISS, while in ASCOT the AP score is used in place of ISS (6).

The Regression Formula and the Corresponding Coefficients used in TRISS (*Table 9*) as Follow: 

Ps=1 / (1+ e-b)

b=b_0_+b_1_ (RTS)+b_2_ (ISS)+b_3_ (Age Index)

As noted above, the coefficients b0–b3 is derived from multiple regression analysis of the Major Trauma Outcome Study (MTOS) database. The Age Index is 0 if the patient is below 54 years of age or 1 if the patient is 55 years and over. The coefficients b0 to b3 are different for blunt and penetrating trauma. If the patient is younger than 15, the blunt index for b3 (Age) is used regardless of mechanism (9). These methods seem promising in quantitative studies of trauma research regarding the age factor, type of trauma, and measures of injury severity.

**Table 9.** The Probability of Survival According to the Trauma and Injury Severity Score (TRISS)

Type of Trauma and the Coefficients	Blunt	Penetrating
b0 ^a^	-0.4499	-2.5355
b1 ^a^	0.8085	0.9934
b2 ^a^	-0.0835	-0.0651
b3 ^a^	-1.7430	-1.1360

^a^ b0-b3, Co efficient indexes

An observational cohort study assessed whether Standardized Assessment of Concussion (SAC) scores and graded symptom checklist scores correlate with symptom severity in children with minor trauma brain injury (mTBI) and with other indicators of mTBI severity, including loss of consciousness and concussion grade (10). In this study, SAC and a graded symptom checklist scores of 348 children aged 6 to 18 years who presented at an emergency department (ED) with blunt head injury (case-patients) and minor extremity injury (controls) were compared. Among case-patients, SAC and graded symptom checklist scores were also compared to American Academy of Neurology (AAN) concussion grades and with the occurrence of loss of consciousness and presence of posttraumatic amnesia. There was a non-significant trend for SAC scores to be lower, reflecting worse cognitive deficits in case-patients relative to controls; however,case-patients had significantly higher graded symptom checklist scores than controls, and the presence of altered mental status in case-patients magnified this effect. Of note, the graded symptom checklist scores were positively correlated with post-traumatic amnesia and AAN concussion grade. The graded symptom checklist reliably identified minor trauma brain injury (mTBI) symptoms for all children aged 6 years and older, as noted by the authors. SAC scores tended to be lower for case-patients compared to controls but did not reach significance. Patients with altered mental status at the time of injury manifest an increased number and severity of symptoms. The authors conclude, "We have demonstrated that the graded symptom checklist within the SAC systematically identifies the symptoms of mTBI in a school-aged pediatric population.... Future efforts should focus on creating a rapid, easily administered tool for detecting the cognitive effects of mTBI in children that accounts for developmental differences and provides an assessment of the likelihood for developing post-concussive syndrome" (10). A system of classification is used in the management of dental trauma in pediatrics, as shown in *Table 10. *

**Table 10.** The Ellis’ Classification for Dental Trauma in Pediatrics: Ellis’ Classification

Simple fracture of the crown involving little or no dentin	Class I
Extensive fracture of the crown involving considerable dentin but not the pulp	Class II
Extensive fracture of the crown with exposure of the pulp	Class III
A fracture in which the entire crown has been lost	Class IV

Other systems of assessment of injury indicating the severity of trauma are summarized in *Table 11. *

**Table 11.** Differential Comparison System for Assessment of the Severity of Trauma and Injury

Initials	Assessment System	Invented Date		Scoring Field	Application Fields	The Basic Criteria
GCS				3–15		Physiologic
	Glasgow Coma Score				Brain injury	Best eye response
						Best verbal response
						Best motor response
OIS of the AAST	Organ Grading Scales of the Committee of the American Association for the Surgery of Trauma	1987		1–6	Surgery of trauma organs injury and their severity	Contusion Stretch injury
						Hematoma
						Laceration
						Perforation
						Disruption
						Transection
						Fracture
						Devascularization
AIS	Abbreviated Injury Scale	1969		1–6	Motor vehicleinjuries	Anatomical
ISS/ NISS	Injury Severity Score/ New Injury Severity Score	1974		0–75	Overall score for patients with multiple injuries	Anatomical OIS of 6 body regions: Head, Face, Chest, Abdomen, Extremities (including Pelvis), External
AP	Anatomic profile				Overall score for patients with multiple injuries- including multiple injuries within 1 body region	Anatomical AP ^a^ of 6 body regions: A- head/brain and spinal cord; B-thorax and front of neck; C-all body regions; D- others
RTS	Revised Trauma Score	1981		0–7/8408	Predicting deathby probability of survival	Physiological:
						Glasgow Coma Scale
						Systolic blood pressure
						Respiratory rate
ICISS	International Classification Of Diseases Injury Severity Score				Injury Severity Score	Survival risk ratio assigned to each ICD-10 code multiplied
TRISS	Trauma Score – Injury Severity Score			b0–b3 ^b^	Determines the probability of survival (Ps) of a patient	ISS ^a^, RTS ^a^, age, and the mechanism of injury (blunt/penetrating)
APACHE	A Severity Characterization of Trauma measure					IAP ^a^, RTS ^a^, age, and the mechanism of injury(blunt/penetrating)
SAC	Standardized Assessment of Concussion				Symptoms of minor trauma brain injury (mTBI) in a school-aged pediatric patient	Concussion grade
AAN	American Academy of Neurology concussion grades				Altered mental status, cognitive deficits, and posttraumatic amnesia	Concussion grade
IIS	Injury Impairment Scale				Focus on non-fatalinjury outcome assessments	The resultant disability
FCI	Functional Capacity Score				Focus on non-fatalinjury outcome assessments	The resultant disability
Ellis’ classification					Management of dental trauma in pediatrics	Tooth fracture, ankylosis luxation, intrusion, dilaceration, root fractures, avulsion

^a^ Abbreviations: AP, Anatomic profile: ISS, Injury severity score; RTS, Revised trauma score

^b^ b0-b3, Co efficient indexes

### 3.2. Prognosis of Injuries

One major reason for the development of new emerging systems of trauma assessment and scoring is to obtain a more precise and accurate estimate of the prognosis of the outcome of injuries, and to provide the corresponding medical care and interventions by creating more reliable practice guidelines. Assessment of the severity of trauma and the extent of related injuries do not always follow a predictable pattern and vary with other factors such as age, anthropometric parameters, and the patient’s physiological reserve functions. The immediate factors of any type of trauma (blunt, penetrating, or explosive) and the scores of assessment are neither so accurate nor remain unchanging as to successfully predict the long-term course of prognosis. The initial care and latent factors should be considered in the management of trauma and it would be beneficial to include these factors in a more comprehensive longitudinal research study of trauma. Furthermore, measures related to patient safety and medical errors that occur in the line of medical services rendered after trauma should be taken into consideration in trauma research. Therefore, a category of factors unique to each case of trauma and injury should be regarded. Review studies of more than 100 articles regarding the use of biomarkers and prospective studies in the research of brain trauma have shown that among all biomarkers (including spectrin-100B, amyloid beta, C tau, neuron specific enolase, and etc.) only the spectrin S-100B breakdown product has acceptable forecasting abilities regarding the later outcomes of trauma under the conditions of the study (11). Studies regarding the cellular and molecular changes in trauma, the effects of drugs and other medical interventions, and the following bodily physiological repair mechanisms are ongoing (12). The clinical parameters of the GCS system are used for this purpose; however, the great variation in the method and lack of a comprehensive approach in these studies has made it difficult to obtain a defined model (4). In some of the reported cases, the response of eye pupils to light has shown useful predictive value for the outcomes of trauma in patients with either low or high GCS scores (5). It is likely that other psychosocial, cognitive, occupational, and personality factors affect the outcome of trauma and the prognosis of its management, hence local and case-specific models would be important tools for trauma research centers.

## 4. Intervention and Follow-up

### 4.1. Investigating the Expected Effects of Interventions After Trauma

The framework of intervention in this model is similar to that of the guidelines for the essential trauma care project (ETC) proposed by the world health organization (WHO). Its primary goal is to assure optimal care of the injured patient across the range of health facilities everywhere, from rural health posts (health houses) whose staff do not have training as doctors, to health network centers staffed by general practitioners, hospitals staffed by specialists (specialist-staffed hospitals), and tertiary care centers, while taking into account the varying resource availability across the spectrum of low- and middle-income individuals. The next step for the research work in our model focuses on performance improvement. These guidelines are designed primarily for health care planners, administrators, other clinicians, and health workers that are involved with the trauma team as well. Guidelines for essential trauma care are crucial, as during their development it was reported that the authors sought to define inexpensive, feasible, minimal standards that would be applicable virtually everywhere in the world (1). These individuals also sought to identify ways of reinforcing existing systems of trauma care in all locations in the world, including the spectrum of conditions found in both low- and middle-income areas. In this process, a list of medical goals was developed that would be feasible for most injured individuals everywhere. These can be viewed as the “needs of the injured patient.” To achieve these goals, the input of human and physical resources in the form of a template must be utilized according to best practices to ensure the best possible outcome. The essential trauma care interventions are categorized into 3 broad sets of needs:

1) Life-threatening injuries are appropriately treated, promptly and in accordance with appropriate priorities, so as to maximize the likelihood of survival.

2) Potentially disabling injuries are treated appropriately, so as to minimize functional impairment and to maximize the return to independence and participation in community life.

3) Pain and psychological suffering are minimized.

Within these 3 categories, there are several specific medical goals that are highly achievable and are subcategorized in Table 12.

**Table 12.** Goals Addressed by the Guidelines for Essential Trauma Care Project

Pain and Psychological Suffering	Potentially Disabling Injuries	Life-threatening Injuries
Medications for the services and for the minimization of pain are readily available when needed	Potentially disabling extremity injuries are corrected	Obstructed airways are opened and maintained before hypoxia leads to death or permanent disability
	Potentially unstable spinal cord injuries are recognized and managed appropriately, including early immobilization	Impaired breathing is supported until the injured person is able to breathe adequately without assistance.
	The consequences to the individual of injuries that result in physical impairment are minimized by appropriate rehabilitative services	Pneumothorax and haemothorax are promptly recognized and relieved.
		Bleeding (external or internal) is promptly stopped
		Shock is recognized and treated with intravenous (IV) fluid replacement before irreversible consequences occur
		The consequences of traumatic brain injury are lessened by timely decompression of space occupying lesions and by prevention of secondary brain injury
		Intestinal and other abdominal injuries are promptly recognized and repaired
		

There are 14 categories of trauma care, each with a basic resource and brief explanation of the rationale used in determining which elements of care are considered essential or desirable. The precise procedures of the interventions referred to as knowledge and skills should be recorded in reference to the above goals, along with the available human and physical resources that are used under the list of equipment and supplies, as well as the items needed for optimal performance of these procedures. The most appropriate method of research to be employed at this stage is either a case study or a before-and-after study, as they are the most useful study methodologies that are used quite frequently in injury research. The value of such studies lies in the usefulness of the findings and suggestions in the preparation, development, or improvement of practice guidelines for the case, the elucidation of clinical or critical pathways involved, and establishment of a clinical protocol that is appropriate to the specific care required. 

### 4.2. Development of Conclusive, Evidence-Based Suggestions or Guidelines

Practice guidelines assist practitioners in making decisions regarding appropriate health care for specific clinical circumstances; however, they are not standards or rules. Guidelines can be as simple or as detailed as a managed care organization deems necessary to provide proper care to members and to consistently monitor the quality of care provided (13). In this respect, it is important to understand that the application of specific guidelines are not required, but are just suggestions in planning methods for improving clinical processes or increasing the cost effectiveness and appropriateness of trauma care. The spoken language is important in the preparation of assessment forms and guidelines and should be considered in exclusion criteria. Research provides information about the need for, the improvement of, and the effects of programs and policies in trauma; evaluative research and mixed method research, more than 40 different studies of which have been reported, contains promising new information to learn and employ.

## 5. Discussion

Six stages of a complete and comprehensive trauma research study may be considered at 3 levels: primary, intermediate, and advanced categories. At each stage, the main operational activities are sequenced in a way that provides the necessary themes for a conclusive study if it is necessary to discontinue, depending on the design of the study being carried out and the presumptive biases. The focus of study should be the significant events, including the trauma and medical care interventions, obtaining information about the subject (s) before and after trauma, and any improvements that can be made to the management of trauma and the follow-up period after treatment. Though not as strong as a randomized, controlled trial (RCT) in establishing a cause and effect study, the low cost, convenience, simplicity, and fewer problems of randomization and ethical concerns are the advantages of these methods. This generic model for the research in trauma should prove helpful, and is shown in *Table 13*. 

**Table 13.** Level, Stages, and Utility Value of Research Plans in Trauma

	Stages of Research in Trauma	Operational Stages	Utility Value (Efficiency & Effectiveness)
Primary			
	First	Diagnosis of the site of injury	Minimum
	Second	Definition of the type of trauma	Restricted
Intermediate			
	Third	Determining the severity of trauma and the magnitude of the injury(ies)	Large
	Fourth	Prediction of the prognosis (forecasting)	Extensive
Advanced			
	Fifth	Assessment of the expected effects of the interventions	Comprehensive
	Sixth	Development of conclusive, evidence-based suggestions or guidelines for prevention and appropriate care and medical services	Complete

Every case of trauma and injury should be taken under study; hence, a case study would be the initial step in the study design.

Considering all of the factors needed in a comprehensive trauma study, there are 5 categories of information that should be temporally distinguished as follows:

a) Information about the subject before the trauma

b) Information about the trauma

c) Information about the subject and the injuries after the trauma

d) Information about the intervention (medical care and treatments)

e) Information about the subject after the intervention

In selecting the most appropriate trauma research design, the time, source of data, and the method of data gathering and analysis are significant factors to consider. It is important to take into account the time frame with respect to the incidence of trauma and the intervention that is applied. This consideration would aid trauma investigators in distinguishing between observed changes due to time trends and changes due to trauma and intervention. The type of information that is collected including the site of trauma, injury, and management as well as the best sources of information and data can be determined in the study design. One may also conduct long-term before-and-after studies or a detailed prolonged case study as comprehensive interventional studies in trauma research. Reports of these studies may be abstracted and outlined, including the following elements:

• Study design

•Definition of injury

•Data sources

•Severity of injury

•Population

•Bias

•Findings
